# *α*-Tocopheryl succinate sensitises a T lymphoma cell line to TRAIL-induced apoptosis by suppressing NF-*κ*B activation

**DOI:** 10.1038/sj.bjc.6600683

**Published:** 2003-01-28

**Authors:** H Dalen, J Neuzil

**Affiliations:** 1 Department of Pathology, The Gade Institute, University of Bergen, Norway; 2 Department of Pathology II, Faculty of Health Sciences, University Hospital, Linköping, Sweden; 3 School of Health Sciences, Griffith University, Southport, Queensland, Australia

**Keywords:** apoptosis, vitamin E succinate, TRAIL, NF-*κ*B, signalling

## Abstract

Activation of nuclear factor-*κ*B (NF-*κ*B) can interfere with induction of apoptosis triggered by the tumour necrosis factor-related apoptosis-inducing ligand (TRAIL; Apo2L). Therefore, agents that suppress NF-*κ*B activation may sensitise cells to TRAIL-dependent apoptosis. Exposure of Jurkat cells to TRAIL resulted in massive and saturable apoptosis induction, following an initial lag time. This lag was abolished by pretreatment of the cells with subapoptotic doses of *α*-tocopheryl succinate (*α*-TOS) or the proteasome inhibitor MG132. Exposure of the cells to TRAIL led to a rapid, transient activation of NF-*κ*B, a process that was suppressed by cell pretreatment with *α*-TOS or MG132. Activation of NF-*κ*B by TNF-*α* prior to TRAIL exposure increased resistance of the cells to TRAIL-mediated apoptosis. We conclude that *α*-TOS sensitises cells to TRAIL killing, at least in some cases, through inhibition of NF-*κ*B activation. This further supports the possibility that this semisynthetic analogue of vitamin E is a potential adjuvant in cancer treatment, such as in the case of TRAIL-mediated inhibition of cancer.

The tumour necrosis factor-related apoptosis-inducing ligand (TRAIL, Apo2L), a member of the TNF superfamily, is a recently discovered potent inducer of apoptosis produced by cells of the immune system (Wiley *et al*, 1995). TRAIL transmits its proapoptosis signal via crosslinking its cognate receptors, death receptor-4 (DR4), also called TRAIL receptor-1 (TRAIL-R1) and DR5 (TRAIL-R2) (Pan *et al*, 1997b; Schneider *et al*, 1997a; Sheridan *et al*, 1997). These receptors recruit and activate the proximal caspase-8, which in turn activates the effector caspases, an event culminating in cell death (Muhlenbeck *et al*, 1998; Bodmer *et al*, 2000; Hopkins-Donaldson *et al*, 2000). The interaction between TRAIL receptors and the proximal caspase is likely mediated by a protein containing the Fas-associated death domain (FADD) (Kischkel *et al*, 2000; Sprick *et al*, 2000) and/or via a GTP-binding adaptor protein (Miyazaki and Reed, 2001). While mitochondrial signalling in TRAIL-induced apoptosis has been postulated in some reports (Thomas *et al*, 2000; Munshi *et al*, 2001), it may not be involved at all in some cases (Walczak *et al*, 2000; Keogh *et al*, 2000), or may act rather as an amplification loop (Suliman *et al*, 2001; Alleva *et al*, 2001).

Two additional receptors for TRAIL have been identified, decoy receptor-1 (DcR-1) (also known as TRAIL-R3) and DcR-2 (TRAIL-R4) (Pan *et al*, 1997a; Schneider *et al*, 1997a,1997b; Sheridan *et al*, 1997; Ashkenazi and Dixit, 1999). These receptors bind TRAIL but fail to transmit its apoptosis-inducing signal downstream, thereby acting as competitive inhibitors of TRAIL apoptosis (Ashkenazi and Dixit, 1999). While the decoy receptors appear unique to the TRAIL system, apoptosis induced by this ligand can also be suppressed by inhibition of caspase-8 (FLICE) activity, via induction of the FLICE-inhibitory protein (FLIP) (Schneider *et al*, 1997b). The fact that normal cells, compared to malignant cells, appear to overexpress DcR-1 and DcR-2 and/or FLIP suggest that TRAIL-induced apoptosis may be selective for cancer cells (Bonavida *et al*, 1999; Kim *et al*, 2000), making TRAIL attractive as a potential anticancer agent (Nagane *et al*, 2001). Furthermore, recent studies indicate that anticancer chemotherapeutics can sensitise cells to killing by immunological agents, including TRAIL, by upregulating the cognate death receptors and/or overcoming TRAIL resistance (Bonavida *et al*, 1999; Nagane *et al*, 2001; Nimmanapalli *et al*, 2001; Matsuzaki *et al*, 2001).

We and others have found that certain analogues of vitamin E, in particular *α*-tocopheryl succinate (*α*-TOS), are potent inducers of apoptosis in a variety of cells (Fariss *et al*, 1994; Neuzil *et al*, 1999,2001d; Yu *et al*, 1999,^2001^), and that this action appears to be specific for malignant cells (Neuzil *et al*, 2001c). Several reports suggest that vitamin E analogues sensitise cancer cells to killing by agents like Fas (Yu *et al*, 1999) or 5-fluorouracil (Chinery *et al*, 1997). As *α*-TOS is an inhibitor of activation of the nuclear factor-*κ*B (NF-*α*B) (Suzuki and Packer, 1993; Erl *et al*, 1997; Neuzil *et al*, 2001b) and because activation of NF-*κ*B has been shown to negatively modulate TRAIL-dependent apoptosis in multiple cancer cells (Bernard *et al*, 2001; Franco *et al*, 2001; Oya *et al*, 2001; Kreuz *et al*, 2001), we have investigated whether this vitamin E analogue might sensitise malignant cells to TRAIL killing via an NF-*κ*B inhibitory activity. In this report, we show that *α*-TOS inhibits NF-*κ*B activation in Jurkat T lymphoma cells and that this amplifies their susceptibility to TRAIL.

## Materials and methods

### Cell culture and treatment

Jurkat T lymphoma cells were maintained in RPMI-1640 medium supplemented with 10% FCS and antibiotics. The cells were regularly split when reaching a density of 1.5×10^6^ ml^−1^, and used for experiments at 0.5×10^6^ ml^−1^. Cells were treated with *α*-tocopherol (*α*-TOH) or *α*-tocopheryl succinate (*α*-TOS) (both from Sigma) at 25 or 50 *μ*M, 10 *μ*M hydrogen peroxide (Fluka) or 40 ng ml^−1^ recombinant human TNF-related apoptosis-inducing ligand (rhTRAIL) prepared as described elsewhere (Alleva *et al*, 2001; Plasilova *et al*, 2002). In brief, the extracellular part of human TRAIL (AA 95-281), obtained by PCR from the HPB T cell line cDNA library, was subcloned into pBSK, sequenced and further subcloned into the His-tagged reading frame of pET15b. The protein was expressed in *Escherichia coli* and purified using the TALON (Clontech) and SP-Sepharose columns. In some cases, cells were treated with the proteasome inhibitor MG132 (Calbiochem) at 0.5 or 1 *μ*M, or tumour necrosis factor-*α* (TNF-*α*; PharMingen) at 100 U ml^–1^.

### Apoptosis assessment

Apoptosis was routinely assessed by the annexin V-binding method, which is based on the affinity of annexin V for phosphatidylserine externalised to the outer leaflet of the plasma membrane early in the course of opoptosis. In brief, cells were harvested by centrifugation, washed with PBS, spun down again, and resuspended in the binding buffer (10 mM Hepes/NaOH, 140 mM NaCl and 25 mM CaCl_2_, pH 7.4). Cells were then incubated with 2 *μ*l of annexin V-FITC (PharMingen) for 20 min at room temperature, and analysed by flourescence-assisted cell sorting (FACS; Becton Dickinson). Activation of caspase-3 was estimated by incubating cells with an anticaspase-3 IgG (PharMingen) that recognises the activated form, followed by incubation with FITC-conjugated secondary antibody. Fluorescence intensity of the cells was assessed by FACS (Neuzil *et al*, 2001b).

### Assessment of NF-*κ*B activation

Activation of NF-*κ*B was estimated using the Trans-AM kit (Active Motif, Carlsbad, CA, USA) according to the manufacturer's protocol. In brief 5–10×10^6^ cells were treated as specified, and lysed using the buffer provided by the manufacturer. The lysates were then transferred into wells containing the immobilised NF-*κ*B (p65) consensus sequence, and incubated for 1 h at 37°C. The wells were washed and the bound p65 protein was detected by horseradish peroxidase (HRP)-dependent staining following incubation with anti-p65 lgG and secondary HRP-conjugated secondary lgG. The level of absorbance at 450 nm, assessed in a microplate reader, reflected the level of bound p65.

### Transmission electron microscopy (TEM)

For TEM, Jurkat cells were grown in complete RPMI medium at 0.5×10^6^ ml^−1^, and treated for 12 h with 40 ng ml^−1^ rhTRAIL or buffer alone (control cells). The cells (10^7^) were briefly rinsed with PBS, centrifuged, fixed overnight in 2% glutaraldehyde, and postfixed with 1% OsO_4_ for 1 h. Both fixatives were made up in 0.1 M cacodylate buffer supplemented with 0.1 M sucrose (pH 7.2, 300 mOsmol) and applied at room temperature. After standard dehydration in ascending concentrations of ethanol, the cells were embedded in Epon-812 monomer and polymerised. Ultrathin sections were cut with a diamond knife mounted in a Reichart ultramicrotome, contrasted with uranyl acetate and lead citrate, and examined in a Jeol 1200 EX transmission electron microscope operated at 80 kV (Brunk *et al*, 1995).

## Results and Discussion

**Figure 1:**
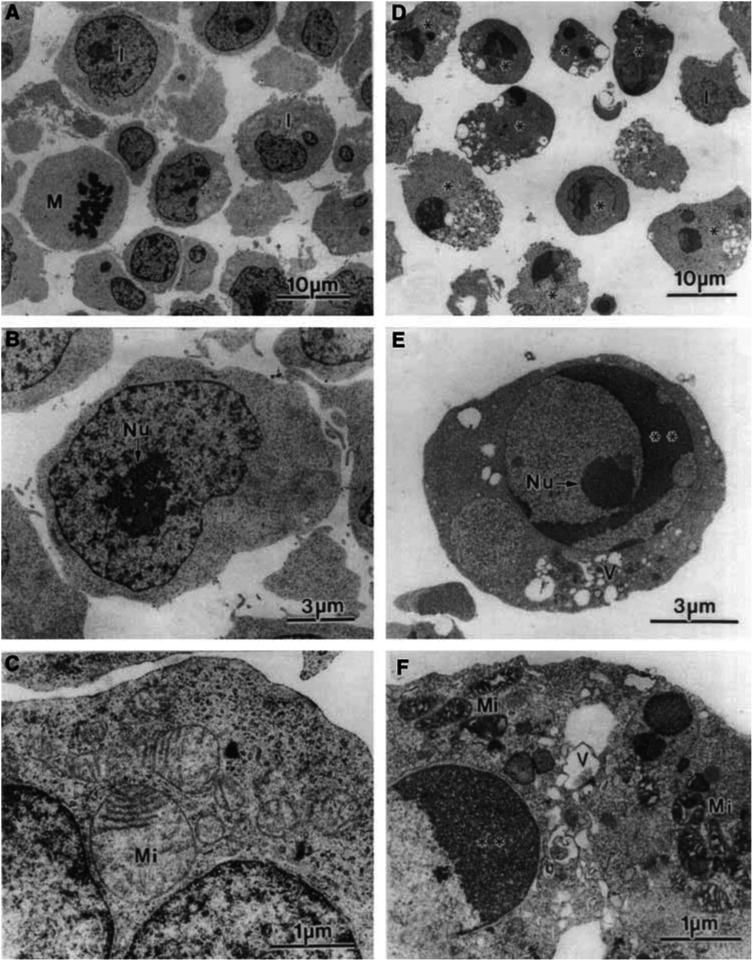
TRAIL is a potent inducer of apoptosis in Jurkat cells. Jurkat T lymphoma cells (0.5×10^6^ ml^−1^) were exposed to the vehicle (**A–C**) or 40 ng ml^−1^ rhTRAIL (**D–F**), processed for TEM, observed, and images taken at the following magnifications: **A, D**–1800×; **B**–5000×; **C, F**–18 000×; and **E**–7000×; M-mitotic cell; I–cell in the interphase; Mi–mitochondrium; Nu–nucleolus; V–vacuole; ^*^apoptotic cell; ^**^nucleus with condensed chromatin.

The aim of the present study was to determine whether the semisynthetic vitamin E analogue, *α*-TOS, could enhance the sensitivity of Jurkat T lymphoma cells to the induction of apoptosis by the immunological agent TRAIL. For initiation of apoptosis, we used rhTRAIL that was expressed in bacterial cells. As shown in Figures 1

and 2

**Figure 2:**
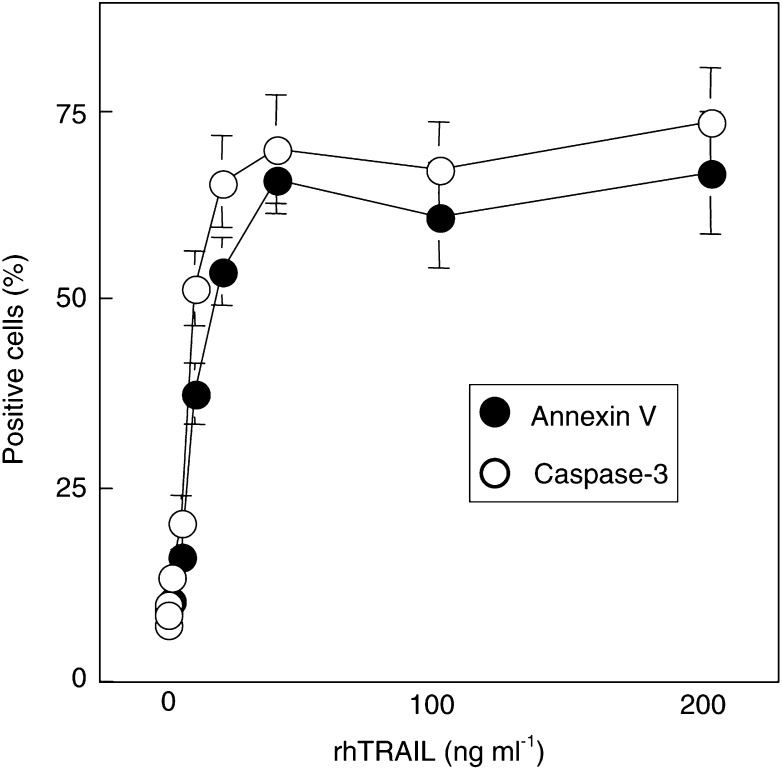
Apoptotic effect of rhTRAIL on Jurkat cells is saturable. Jurkat cells at 0.5×10^6^ ml^−1^ were exposed to increasing concentrations of rhTRAIL for 12 h, and the extent of apoptosis (annexin V-FITC staining) and caspase-3 activation was assessed.

, our rhTRAIL preparation caused massive apoptosis in Jurkat cells, as documented by both morphological changes evaluated by TEM, and by PS externalisation and caspase-3 activation. Figure 2 also demonstrates that apoptosis induction was saturable with regard to the rhTRAIL used, with rhTRAIL being maximally effective at ca 20 ng ml^−1^.

**Figure 3:**
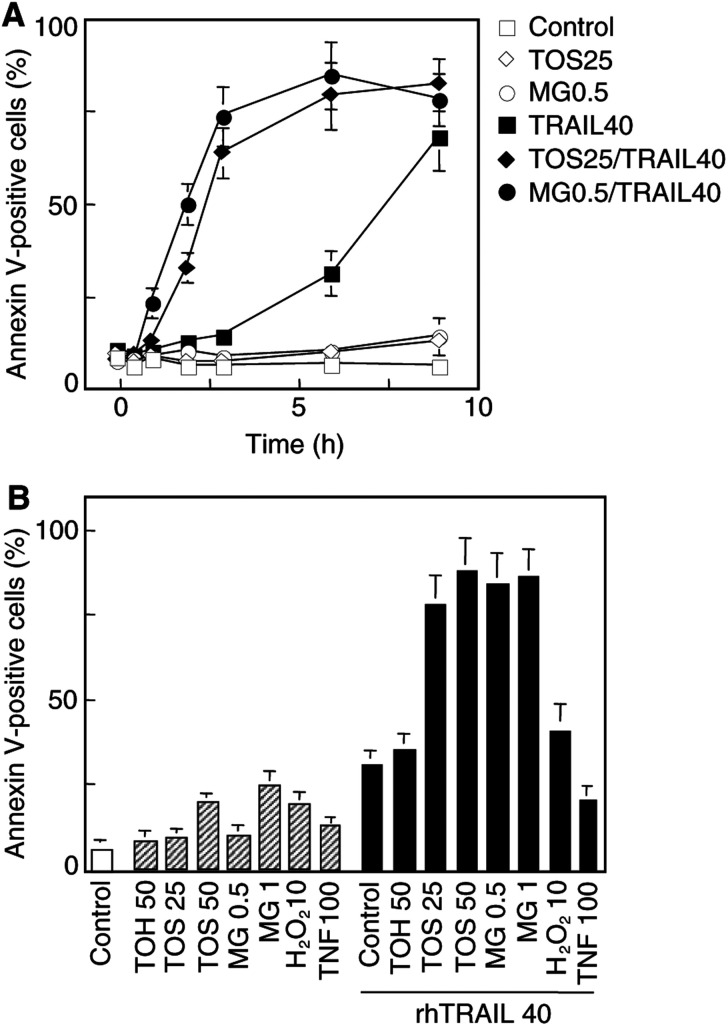
*α*-TOS sensitises Jurkat cells to TRAIL killing. Jurkat cells (0.5×10^6^ ml^−1^) were pretreated with *α*-TOH, *α*-TOS, MG132 or hydrogen peroxide at the concentrations indicated (*μ*M) for 4 h, or to TNF-*α* at 100 U ml^−1^ for 1 h, after which they were exposed to rhTRAIL at 40 ng ml^−1^. At time points indicated **(A)** or at 6 h **(B)** following TRAIL addition, cells were evaluated for apoptosis.

We next investigated whether preincubation with *α*-TOS might sensitise Jurkat cells to TRAIL. As shown in Figure 3

, *α*-TOS–but not *α*-TOH–rendered the cells more susceptible to TRAIL-induced apoptosis at a concentration at which the vitamin E analogue itself did not cause substantial cell death. To determine whether this potentiation of TRAIL killing might invlove inhibition, by *α*-TOS, NF-*κ*B activation, the cells were pretreated with the proteasome inhibitor, MG132, or with TNF-*α*, a potent activator of NF-*κ*B. Figure 3 shows that preincubation with MG132 sensitised cells to TRAIL, as did *α*-TOS, and that MG132 itself did not cause apoptosis. On the contrary, pretreatment with TNF-*α* increased resistance of the cells to TRAIL, consistent with the idea that activation of NF-*κ*B may be antiapoptotic (Bernard *et al*, 2001; Franco *et al*, 2001). Finally, we used hydrogen peroxide as a negative control. At a low concentration (10 *μ*M) that does not interfere with NF-*κ*B activation (see below), hydrogen peroxide did not induce substantial apoptosis nor did it sensitise cells to TRAIL (Figure 3).

**Figure 4:**
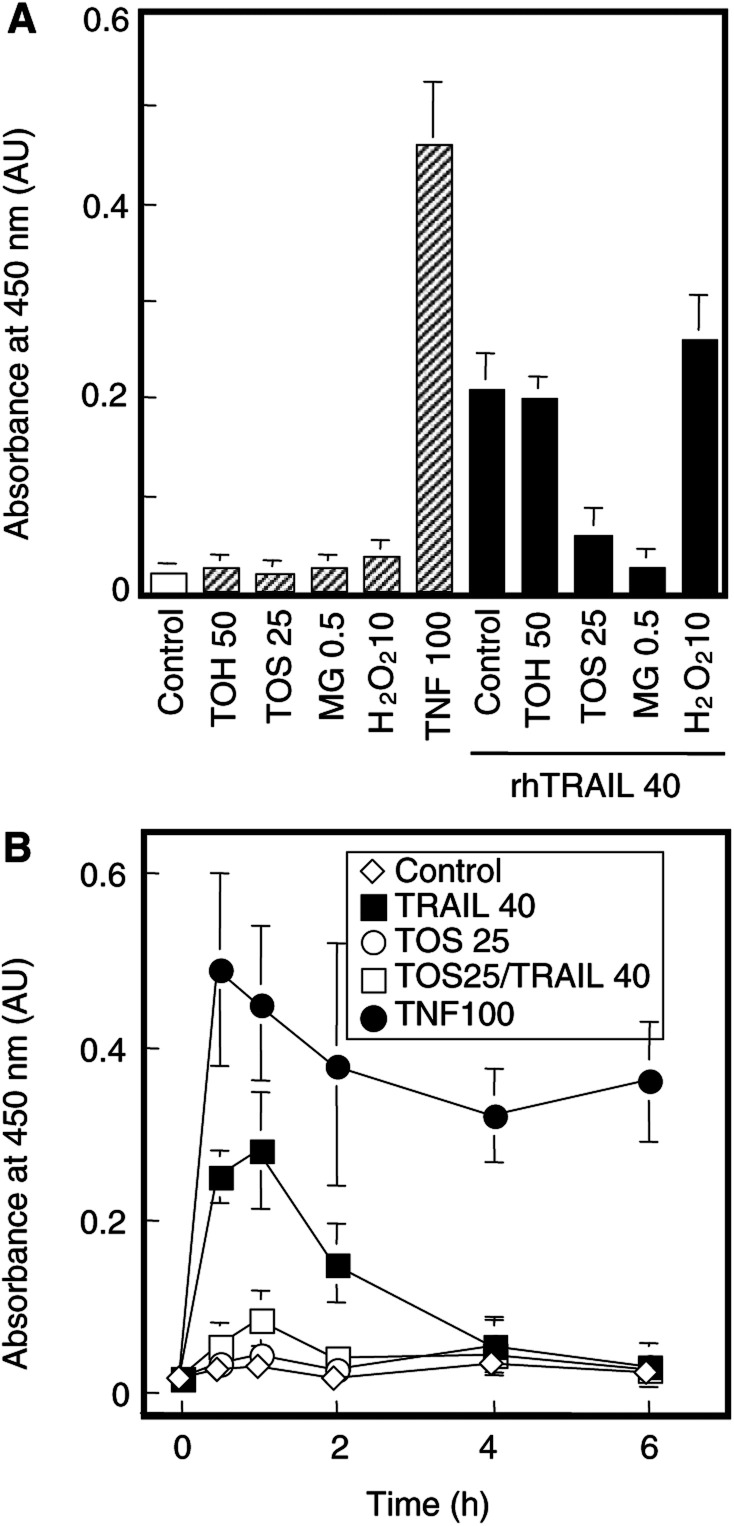
*α*-TOS abolishes transient activation of NF-*κ*B by TRAIL. Jurkat cells (0.5×10^6^ ml^−1^, 10^7^ total) were treated as specified in the legend to Figure 3 (concentrations in *μ*M except U ml^−1^ for TNF-*α*, rhTRAIL at 40 ng 1^−1^). At a 2-h time point **(A)** or as specified **(B)**, cells were washed with PBS, spun down, the pellet resuspended in the lysis buffer, and the lysate probed for NF-*κ*B activation using the TRANS-AM kit as detailed in Materials and Methods. The level of NF-*κ*B activation (p65 binding to its cognate DNA sequence) is expressed as a relative absorbance at 450 nm.

More direct studies of NF-*κ*B activation revealed that Jurkat cells exposed to rhTRAIL did activate NF-*κ*B. Figure 4

shows a substantial activation of NF-*κ*B, 30 min following addition of rhTRAIL to the cells, although this activation was less pronounced than that caused by treatment with the strong NF-*κ*B activator, TNF-*α*. This activation was transient and lasted for about 1 h, after which it declined. Pretreatment with *α*-TOS or MG132 abolished the initial NF-*κ*B activation observed with TRAIL alone. Once again, hydrogen peroxide at 10 *μ*M had no effect on NF-*κ*B, either alone or in combination with TRAIL (Figure 4).

We demonstrate in this communication that vitamin E succinate, but not vitamin E itself, potentiates killing of Jurkat T lymphoma cells by the immunological inducer of apoptosis, TRAIL. These data are consistent with, and further extend, the earlier observations that *α*-TOS promotes apoptosis caused by a variety of agonists. This is true, for example, of Fas-dependent killing of breast (Yu *et al*, 1999) and prostate cancer cells (Israel *et al*, 2000). In these instances, the vitamin E analogue sensitised the cells to Fas ligand by causing plasma membrane translocation of Fas. Furthermore, *α*-TOS also promotes TRAIL-induced apoptosis in colon cancer cells, apparently by modulating different, converging signalling pathways, thereby maximising the apoptotic potential of the cells (Weber *et al*, 2002). Importantly, this cooperation was also reflected in the inhibition of colon cancer in an animal model (Weber *et al*, 2002).

There are several possible mechanisms by which *α*-TOS may sensitise leukemic cells towards TRAIL killing. TRAIL crosslinks two cognate, death-signalling receptors. One of these, DR4 (TRAIL-R1), has been shown to transiently activate NF-*κ*B. This leads to an initial expression of survival signals including the inhibitor of apoptosis protein (IAP) family members (Degli-Esposti *et al*, 1997; Schneider *et al*, 1997b; Bernard *et al*, 2001). Perhaps by this mechanism, activation of NF-*κ*B can protect leukemic cells from apoptotic killing (Jeremias *et al*, 1998). Activation of NF-*κ*B also leads to upregulation of the caspase-8 inhibitor, cFLIP (Kreuz *et al*, 2001). Jurkat cells express both DR4 and DR5, although the level of expression of the former receptor is lower than that of the latter (JN *et al*, unpublished). In spite of this, the level of DR4 expression appears to be sufficient to activate NF-*κ*B upon exposure of the cells to TRAIL (this report). We hypothesised that inhibition of NF-*κ*B activation–likely responsible for the lag phase in apoptosis induction by TRAIL in Jurkat cells– could be inhibited by *α*-TOS. In support of this, preincubation of the cells with *α*-TOS suppressed TNF-*α*-dependent NF-*κ*B activation (cf. Fig. 4). The exact mode of suppression of NF-*κ*B activation by *α*-TOS is not yet clear but there are several possibilities. For example, activation of NF-*κ*B might be suppressed by *α*-TOS by affecting degradation of the inhibitory subunit, I*κ*B. Indeed, cleavage of, or mutations in, I*κ*B can accentuate apoptosis (Jeremias *et al*, 1998; Keane *et al*, 2000) and a recent report documents a caspase-dependent cleavage of I*κ*B in TRAIL-resistant cells, thereby sensitising them to killing by TRAIL (Kim *et al*, 2002).

The concept that *α*-TOS can inhibit NF-*κ*B activation is not new (cf. Erl *et al*, 1997), but the precise structural requirements are not fully known. It is clear, however, that *α*-TOH, the redox-active counterpart of *α*-TOS, fails to exert such activity (Erl *et al*, 1997; Neuzil *et al*, 2001b). One possibility is suggested by the observation that *α*-TOS activates caspases that cleave the NF-*κ*B subunit p65, while not killing the cells (Neuzil *et al*, 2001b), probably via a mitochondria-dependent pathway (Neuzil *et al*, 2001d; Weber *et al*, 2002). We have earlier suggested that under certain circumstances, *α*-TOS can cause ‘subapoptotic’ signalling that may lead to activation of early apoptotic events while not bringing the cell into the execution phase of apoptosis, a possibility also suggested by others (Harvey *et al*, 2000). Such a mechanism may underlie the inhibitory activity of *α*-TOS towards activation of NF-*κ*B in Jurkat cells, thereby sensitsing the cells to killing by TRAIL.

*α*-TOS is not just another example of an inducer of apoptosis that senstises cells to TRAIL killing, a principle that has been published in multiple reports (see, e.g. Bonavida *et al*, 1999; Nagane *et al*, 2001). Unlike many chemotherapeutic agents, *α*-TOS appears to be selective for malignant cells (Neuzil *et al*, 2001c,2001d; Weber *et al*, 2001). *α*-TOS, which has proapoptotic activity *in vitro* and antineoplastic effects *in vivo* (Neuzil *et al*, 2001d; Malafa *et al*, 2002; Weber *et al*, 2002), is carried within the bloodstream by circulating lipoproteins (Pussinen *et al*, 2000), which are cleared in the liver. Here, *α*-TOS is hydrolysed to *α*-TOH, at least some of which is released into the circulation, thereby boosting the antioxidant defence system (Neuzil *et al*, 2001a). Because both *α*-TOS and TRAIL are relatively nontoxic to normal cells (Bonavida *et al*, 1999; Jo *et al*, 2000; Kim *et al*, 2000; Nagane *et al*, 2001; Neuzil *et al*, 2001c,2001d; Nesterov *et al*, 2002; Weber *et al*, 2002), the two agents, that is, *α*-TOS and TRAIL, would seem to represent an exciting partnership of potentially high therapeutic relevance.

In conclusion, we have shown that *α*-TOS potentiates TRAIL-induced apoptosis in Jurkat T lymphoma cells by inhibiting transient activation of the transcription factor NF-*κ*B. In practical terms, this finding could be utilised for devising strategies of treatment for potentially fatal disorders like lymphomas or carcinomas on two levels: first, by coadministration of *α*-TOS and TRAIL; second, by administration of *α*-TOS alone, as the agent could be expected to sensitise cancer cells to endogenously produced TRAIL, thereby potentiating the immune defences against neoplasia. This principle may be especially useful for suppressing cancer involving malignant cells with a high expression of DR4. Further exploration of these possibilities may lead to an effective approach to the treatment of malignancies.
